# Role of Forensic Odontology in Identification of Persons: A Review Article

**DOI:** 10.7759/cureus.56570

**Published:** 2024-03-20

**Authors:** ِAbdel Naser M Emam

**Affiliations:** 1 Prosthetic Dental Science, Faculty of Dentistry, Najran University, Najran, SAU

**Keywords:** cheiloscopy, recent concepts, denture marking, identification, forensic odontology

## Abstract

Forensic dentistry plays a pivotal role in identifying deceased individuals when visual or other means of identification are not possible, particularly in the aftermath of mass disasters or criminal activities. Accurate and timely identification of the deceased and injured becomes crucial following events like earthquakes, fires, transport accidents, gunshot incidents, floods, tsunamis, bomb blasts, and terrorist attacks. The process of creating a person's identity is a formidable task, often relying on prevalent methods such as dental, DNA, and fingerprint analyses. Forensic odontology, a specialized field within dentistry, assumes a significant role in identifying individuals in accidents, civil unrest, natural and mass disasters, and crimes related to genocide. In cases where natural teeth are absent, the marking or labeling of dentures becomes essential for personal identification. Teeth's resilience to destruction and decomposition makes dental identification feasible even under extreme conditions. The fundamental principle of forensic dentistry rests on the uniqueness of each individual's oral structure, emphasizing that no two mouths are identical, not even in the case of twins. The purpose of this review is to explore the role of forensic dentistry in identifying individuals through various methods such as denture labeling, cheiloscopy, radiographs, bite mark analysis, rugoscopy, salivary signature, age and sex estimation, dental DNA identification, individual characteristics, and denture marking. Based on detailed ante-mortem records from dental specialists, which are compared to postmortem data during investigations, forensic dentistry is a trustworthy technique for identifying deceased individuals and criminals.

## Introduction and background

The history of forensic odontology spans from ancient Rome to the 21st century, with continuous evolution. Dr. Oscar Amoedo is credited as the pioneer of forensic odontology for his groundbreaking work in identifying fire accident victims in Paris [1]. Forensic odontology, a dental branch, involves handling, examining, and presenting dental findings for justice. Accurate identification in disasters or assaults is crucial, often relying on dental factors like restorations, missing teeth, and prosthetic devices when other records are unavailable. Prosthodontics, especially denture identification systems, play a vital role in identifying edentulous individuals’ postmortem. From printed labels to advanced technological methods, including implant-retained dentures and radiographs, prosthodontists contribute significantly to human identification. This literature review highlights the pivotal role of dentists in forensic odontology [2].

Prosthetic dentistry has proven to be crucial in enhancing the accuracy and reliability of investigatory data in forensic science. Various methods, including palatoscopy, implants, cheiloscopy, preprosthetic records of surgery, prosthesis marking systems, and bite marks, contribute to this effort. Ante-mortem dental records encompass a comprehensive set of information, including medical and dental histories, written notes, diagrams, charts, radiographs, test results, clinical photographs, referral letters, study models, prescriptions, and relevant data [3]. Denture identification systems are vital not only for hospitalized and long-term care patients but also for forensic identification purposes and social reasons. In some cases, the only identifiable remains of a victim are partial or complete dentures. Denture marking facilitates the return of lost dentures and aids in the identification of both living and deceased denture wearers. Patients with neurodegenerative disorders, such as Parkinson's disease, characterized by memory and cognitive dysfunctions, can be easily traced through the labeling of their prostheses. Forensic odontologists, International Dental Associations, and US legislation recommend labeling of dentures [4].

Avon categorized forensic odontology into three main areas: (i) civil, (ii) criminal, and (iii) research. The civil domain focuses on mass disasters like earthquakes and airline and train accidents, requiring victim identification amid extensive physical damage. It also tackles issues related to malpractice and fraud, handling claims related to damage, and age assessment without a birth certificate. The criminal field focuses on identifying individuals through dental remains in cases of rape, homicide, and suicide, using techniques like cheiloscopy, analysis of bite marks, and palatal rugoscopy. Finally, the research domain is dedicated to providing training in forensic odontology for healthcare and dental practitioners. Dentists in this field play a vital role by comparing dental features in criminal as well as civil contexts, such as mass disasters, suicide, rape, and reconstructive postmortem cases. This involves the utilization of evidence like lip and bite marks and radiographic data and comparing it with previous records, including photographs, cast models, and dental X-rays [5].

## Review

Awareness of forensic odontology among dentists in Saudi Arabia

Alhazani et al. investigated the awareness of forensic odontology among dental students in Riyadh, Saudi Arabia. Results showed that 75%, 42%, and 40.9% of postgraduates, graduates, and undergraduates, respectively, recognized teeth as a DNA source. Additionally, 95% of participants were aware of forensic dentistry's role in investigating criminals and the deceased. Regarding identification in mass disasters, 77.5% and 72% of postgraduates and undergraduates, respectively, acknowledged forensic odontology's assistance in determining gender and age using dental record data. However, a lack of awareness regarding job prospects in forensic dentistry in Saudi Arabia was noted among 62.73% of undergraduates, with 97.5% revealing that forensic dentistry was not integrated into the curriculum of dental courses [6].

The relationship between forensic odontology and various dental specialties

Thetakala et al. analyzed the intersection of forensic odontology with different dental specialties. The Journal of Forensic Dental Sciences featured a majority of articles from oral medicine and radiology (32.6%), primarily emphasizing cheiloscopy (46.7%). In the Journal of Forensic Odonto-Stomatology, prosthodontics led in article publications (25.7%), with a primary focus on bite mark analysis (66.7%). Overall, oral medicine and radiology contributed the most articles across various dental specialties (28.3%) in both journals, with cheiloscopy being the predominant focus (41.7%). Notably, the Department of Periodontics had no articles in forensic odontology, and limited contributions were observed from conservative dentistry and endodontics [7-8].

Forensic dentists compare ante-mortem records with post-mortem findings to establish positive matches in identifying individuals through various methods

Denture Marking

In cases involving edentulous individuals, various identification methods can be employed, including the comparison of paranasal sinus anatomy and bony patterns observed in radiographs. Additionally, the victim's dentures, often located in their mouth or at home, offer valuable information about denture creation, materials used, and distinctive shapes. This data can serve as important ante-mortem records or postmortem evidence [7].

In Sweden and Iceland, denture marking is subject to legal regulations. In the year 1986, the "National Board of Health and Welfare," the overseeing authority for the health department, mandated all dentists offer patients the option to mark their dentures with a personal number. Additionally, dentists must transparently inform and motivate patients about the advantages of denture marking. The American Dental Association (ADA) has outlined particular denture marking criteria, including specificity, cosmetic acceptability, simplicity of technique, preservation of denture strength, and resistance to fire and solvents. The optimal locations for denture markers, accessible to readers, are the lingual flange of mandibular dentures and the posterior buccal surface of maxillary dentures. Other viable sites include those within the buccal, palate, and tuberosity regions. Critical requirements for any denture identification system include protection against the monomer in denture base resin, resilience to high processing temperatures, consideration of polishing and finishing procedures, and durability against wear and tear over an extended period [2].

Dr. Robert H. Griffiths highlighted the significance of denture identification in forensic odontology. The ADA endorses denture labeling for improved identification. Multiple methods, such as scribing numbers, barcodes, metal bands, lead foil, photographs of patients, electronic microchips, radiofrequency tags, paper strips, and laser-etched discs, are employed for denture marking. This system proves valuable in identifying geriatric individuals, particularly in accidents and mass disasters [9]. Certain ideal characteristics for a denture marker include biological inertness, ease of application, aesthetic acceptability and readability, cost-effectiveness, retrievability after accidents, acid resistance, durability without compromising prosthesis strength, resistance to common disinfecting agents, and the ability to withstand elevated temperatures [10].

Denture marking can be classified into various methods as outlined in the literature

Surface Marking Methods

**Figure 1:**
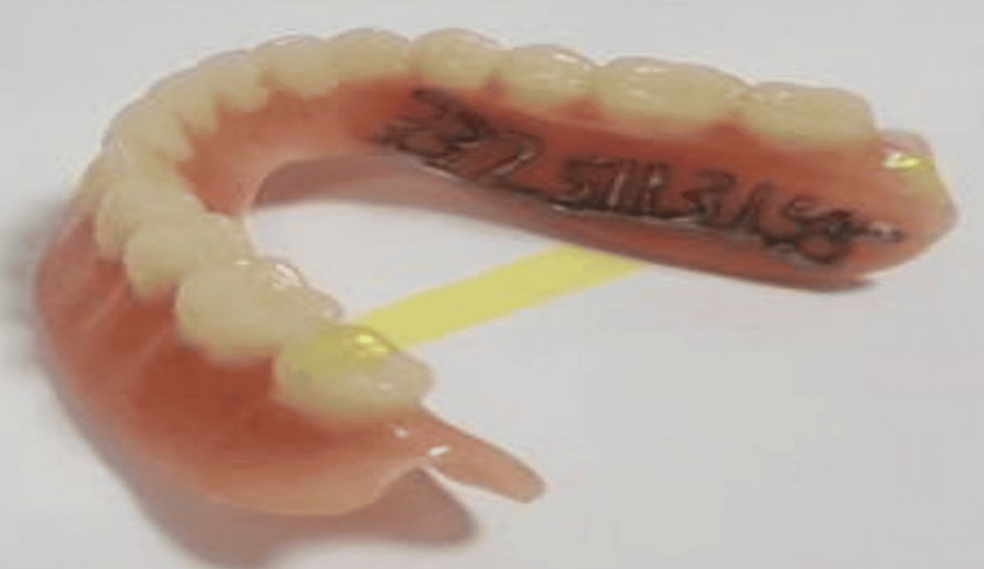
Lower denture displaying the incorporation of a cast metal unique national identification number

Writing on the tissue or polished surface: Information is written directly on the denture's tissue surface or polished surface. *Scribing or engraving method*: In this technique, labeling is directly applied to one of the denture's surfaces by engraving numbers (Figure 1) or letters onto the fitting surface of the maxillary denture using a small round bur. However, there is a potential risk of bacterial infections due to the engraved areas serving as sites for food debris lodgment.

Embossing technique: In this method, the patient's surname and initials are scratched onto the master cast, and these details are subsequently transferred to the intaglio surface of the denture. However, despite being cost-effective, this technique may pose concerns such as infection, food entrapment, and irritation. The presence of embossed letters may result in persistent tissue irritation and, in some cases, could potentially lead to malignancy.

Fiber tip pen marking: Another surface marking method involves using a fiber tip pen to write on the polished or tissue-fitting surface of the finished denture. This approach is considered a temporary marking method.

Fingerprint integration in prosthesis: This innovative approach involves capturing the unique fingerprint of an individual on paper, which is then laminated and incorporated into the prosthesis. Concurrently, digital data of the fingerprint are recorded and then stored. In the event of the need to identify a suspected individual, this type of data can be recovered from the dentures of the patient and coordinated for identification purposes [11].

Inclusion Methods

**Figure 2:**
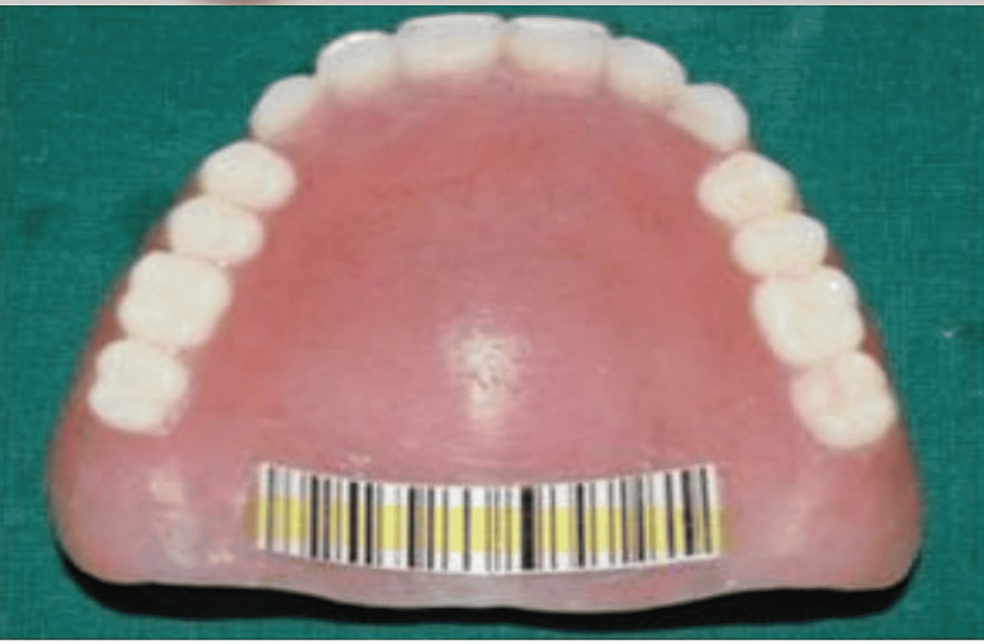
Barcode

Quick response (QR) code: This method involves embedding a QR Code within dentures to store a substantial amount of patient information. The code is encapsulated within the denture structure (Figure 2).

Label enclosure: Labels are enclosed within the denture during the packing stage using materials such as metallic or non-metallic substances, micro labels, and microchips. While these techniques offer greater permanence compared to surface marking, drawbacks include increased time consumption and the potential for issues like dislocation, wrinkling, or tearing during the packing stage. Denture barcoding is a crucial tool for identifying individuals in various scenarios, including accidents, dementia, unconscious states, missing persons, or during natural disasters when identifying deceased bodies is necessary. These barcodes, incorporated beneath a thin layer of clear acrylic in the prosthesis, can be readily scanned using barcode readers or optical scanners. This scanning process retrieves encoded data, facilitating quick access to valuable information about the individual. Additionally, this method allows for the storage of supplementary details, such as photographic, radiographic, and clinical records of the patient [12].

Laser micro-etching: This method involves the use of a copper vapor laser to precisely etch the details of a patient onto the denture. Laser micro-etching proves to be a precise, cost-effective, and promising technique for labeling metallic prostheses. It offers a permanent means of personal identification.

Identification (ID) band: An ID band is made from stainless steel metal and contains information about a patient.

Paper strips: This technique employs a typed strip of onion skin paper to label dentures.

T-shaped bar: An enclosed clear bar made of poly methyl methacrylate resin, shaped like a 'T,' is incorporated into the denture, referred to as a label.

Radio-frequency identification (RFID) tags: RFID tags are embedded within the fitting or the polished surface of an already-present denture.

Lenticular system: This involves utilizing a card system to store patients’ details in an image format, visible by altering the viewing angle. These cards can be integrated into either the palatal or lingual flange of the mandibular denture [13].

Photograph embedding: Patient photographs are embedded in the denture, albeit with a resistance limited to temperatures of 200-300°C.

**Figure 3:**
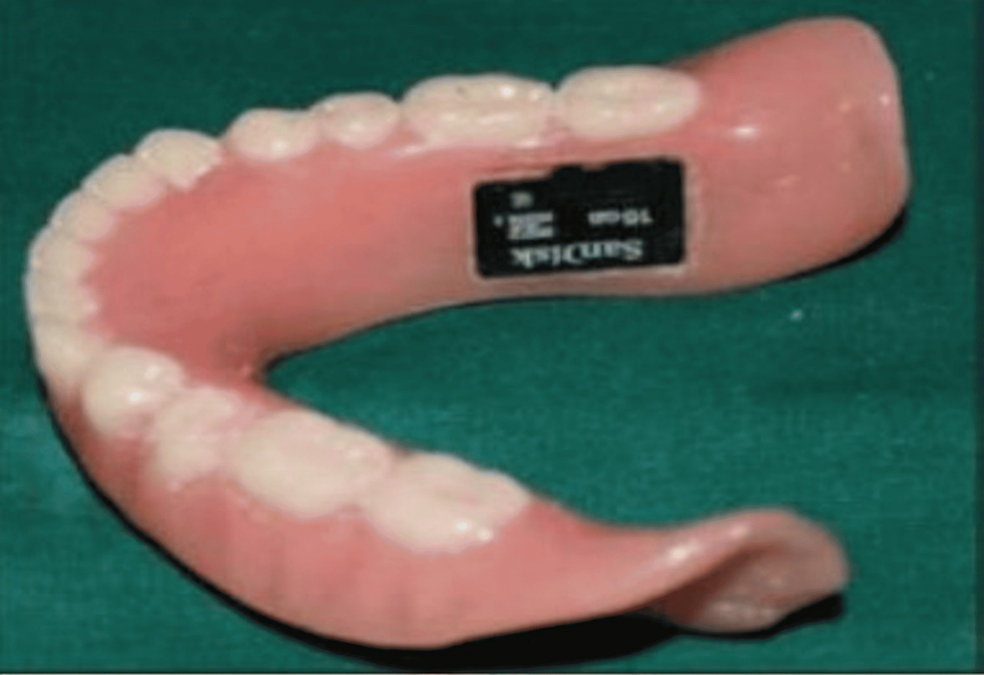
Micro SD card Micro SD: memory cards

Memory (micro SD) cards (Figure 3): Electronic microchips measuring 5 × 5 × 0.6 mm, known as Micro SD cards, are aesthetically acceptable and resilient under high temperatures. These cards, which contain pertinent information, can be integrated into denture flanges by cutting a piece of the denture to match the size of the Micro SD card, followed by covering it with auto-polymerizing acrylic resin. The information can be accessed later as required, facilitating the identification of the deceased. Notably, this method offers a benefit over barcodes, as data retrieval can be performed by any relevant individual when necessary [11].

One significant challenge in identifying individuals arises when they are completely edentulous. Three potential options are available in such cases. The first involves the use of denture markers, while the second utilizes dental implants, each carrying a unique number of records maintained by dentists. The third choice entails obtaining a detailed mouth orthopantomogram for these patients, aiding in the identification of distinctive features related to jaw bone defects, condyle and coronoid positions, and the place of anatomical structures like the mandibular canals and maxillary sinus within the bones of the jaw. Another issue lies in keeping records for individuals with a healthy oral cavity and no dental issues, though it is challenging but feasible. In such instances, the bite's class characteristics can be recorded using study models, which are digitally scanned and saved for future reference [14].

Fixed Dental Prostheses and Implants

Chips micro-sized with digital information can be embedded in tooth structures for individual identification, retrievable by advanced technology. Tooth structures provide robust protection for these chips against high temperatures, acid attacks, and exposure to humid and saline environments. Coded dental implants, with specific numbers assigned to the implant body or abutment, enable individual identification from remains after incidents. It's essential to recognize that this information is limited to a specific commercially available system [15].

Prosthetic rehabilitation using dental implants is a common oral treatment, offering fixed crowns or movable partial/total dentures. The variety of available implant systems presents distinct designs. A comprehensive catalog of radiographic images and implant descriptions would assist in identifying manufacturers and types in forensic cases. When an unidentified body has dental implants and lacks dental records, insights from implant types guide investigations. Analysis of dental porcelain components aids in dental identification, with fluorescence examination lamps used to study victim and control samples. While effective, a faster, non-destructive method for element detection in dental porcelain is needed [16].

Individual Characteristics

In forensic odontology, the identification of victims relies on "preserved dental records" or "ante-mortem records" maintained by general dental practitioners. These records include the size and shape of teeth, root characteristics, tooth placement, the number of teeth, and details about dental procedures such as crowns, extractions, bridges, fillings, and root canals. Comparing ante-mortem and postmortem records proves to be a more reliable and straightforward method for identifying a deceased individual when compared to alternative approaches [17].

Cheiloscopy

Lip print identification, characterized by the analysis of distinctive line arrangements on the red part of the lips, can be described as a technique for recognizing individuals. This methodology closely resembles fingerprint comparison and is widely acknowledged as a valid form of scientific analysis for human identification based on lip traces. The unique patterns found in lip prints are considered to be specific to each individual, making them a valuable resource for identification purposes [18].

Lip prints, formed by elevations and depressions on the lip's external surface, are distinct patterns. Recoverable from various surfaces at crime scenes, such as clothing, cups, and cigarettes, they are globally acknowledged in forensic odontology for personal identification. Essential anatomical landmarks on the lip, including the chelion, stomion, labrale superius, and labrale inferius, contribute to this recognition. These genetic, unique, and permanent lip prints remain unaltered even after death, serving as a dependable method for identification [19].

**Figure 4:**
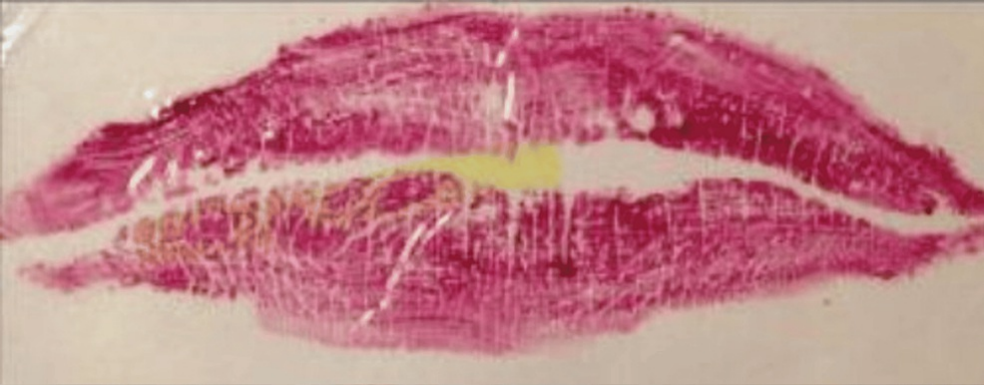
Type 1: distinct vertical grooves spanning the entire lips

**Figure 5:**
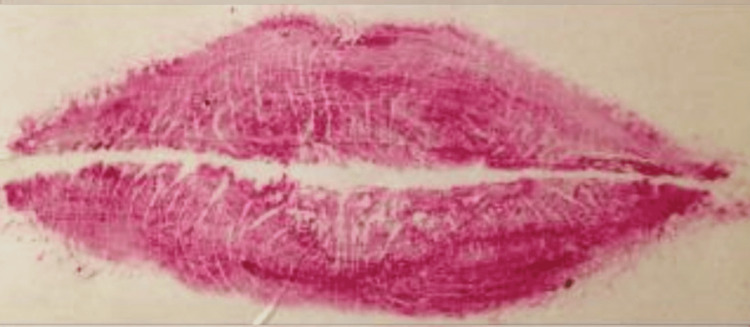
Type 2: grooves with branching patterns

**Figure 6:**
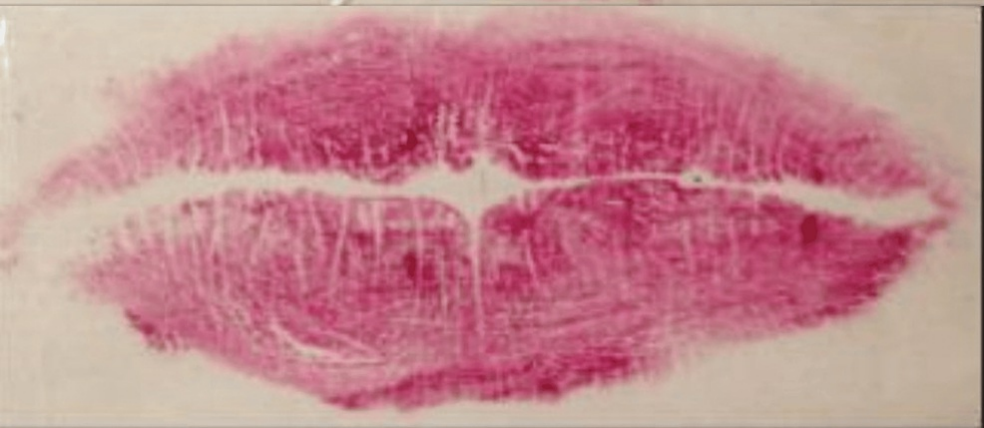
Type 3: grooves that intersect

**Figure 7:**
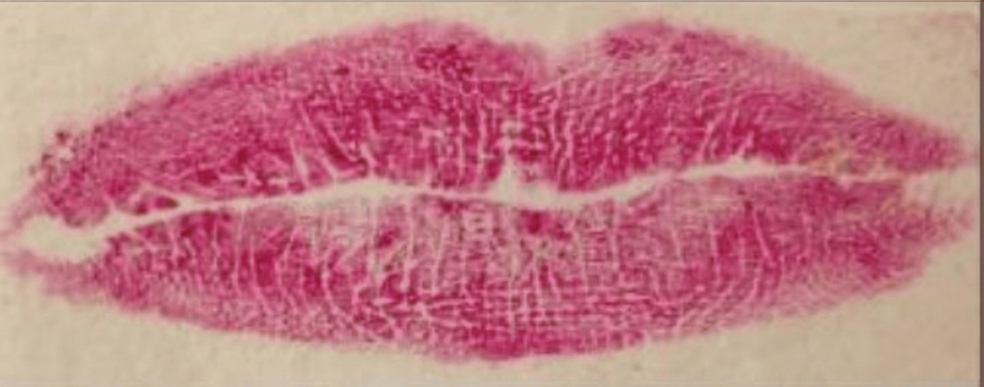
Type 4: reticular grooves

**Figure 8:**
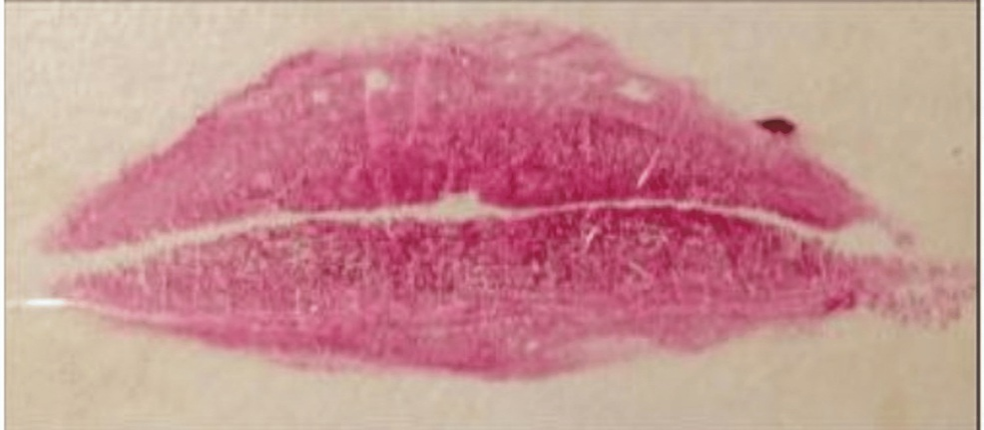
Grooves that do not fit into Types 1–4 and cannot be morphologically differentiated

The categorization of lip line patterns introduced by Suzuki and Tsuchihashi, widely adopted in literature, is as follows [20]: Type 1: clear-cut vertical grooves spanning the entire lips; Type 2: branching groove pattern; Type 3: intersecting grooves; Type 4: reticular grooves; Type 5: other types of grooves that do not fit into Types I-IV and cannot be differentiated morphologically (undetermined) (Figures 4-8).

Recording Lip Prints Involves Two Methods

Application method: Apply lip rouge, lipstick, or another suitable transfer medium to the lips. Have the individual press their lips onto cellophane tape, paper, or a similar surface.

Photography method: Photograph the lips, especially on a flat, non-porous surface like a mirror. Enlarge the photographs and create overlay tracings of the lip grooves. In practice, individuals had their lips cleaned and a dark red lip liner marked on the upper and lower vermilion borders. Using this, a lip outline impression was created on a cellophane tape strip, starting from the center and uniformly pressing toward the lip corners. The cellophane strip, now affixed to white chart paper for a permanent record, was then examined using a magnifying lens. The lip print recording is sensitive to various factors. To ensure accuracy, lip prints should be obtained within 24 hours of death to avoid postmortem alterations. Pathologies like mucocele, surgical changes, and loss of anterior teeth can impact lip print patterns. Additionally, debris, excessive lipstick, or overstretching of cellophane tape may alter recordings. While lip prints are unique, unclear lines make individual identification challenging, unless the trace includes distinctive features like scars and clefts [19].

The distinctiveness of lip patterns relies on the relaxation of lip muscles, generating specific patterns that vary between open and closed mouth states. Well-defined lines are evident in the closed mouth position, while the grooves become less distinct and challenging to assess in the open position. Consequently, only closed-mouth lip prints were considered for analysis [21].

Rugoscopy

Rugoscopy in forensics involves analyzing palatal rugae for personal identification. Palatal rugae refer to the folds of the palatine mucosa positioned laterally to the mid-palatine raphe on the front palate, extending transversely for different lengths. Similar to fingerprints, palatal rugae maintain their morphological consistency throughout an individual's lifetime post-development. The dentition and buccal adipose tissue protect the palatal rugae from deformation caused by trauma or extreme temperatures. These rugae can be well preserved for a few days after death [22]. The proposal to utilize palatal rugae designs for personal documentation was initially introduced in the study of Allen in 1989 [23]. Palatal rugae impressions on maxillary denture surfaces can be compared with those of the deceased using high-definition impression materials, casting, or computerized recording. Typically comprising three to seven ridges arising from the incisive papilla, these rugae patterns, categorized as curved, straight, wavy, or branched, are exclusive to each individual. In cases where dental identification of the postmortem is challenging, especially in edentulous individuals, palatal rugae may serve as an additional method. However, rugoscopy's definitive use in forensic odontology is limited, as it requires existing ante-mortem records for postmortem identification [24].

Palatal rugae patterns serve as an alternative identification method in cases of tooth loss, particularly due to trauma, due to their unique characteristics. Internally positioned within the oral cavity and shielded by the buccal fat pad and tongue, rugae remain resilient to heat and external factors. Changes in palatal rugae patterns may result from factors like aging and environmental factors such as orthodontic procedures, periodontal and cleft palate surgery, tooth extraction, and an affected canine eruption. Rugae analysis methods include photographs, maxillary arch impressions, software programs like RUGFP-ID, overlay print, calcorrugoscopy, stereoscopy for a 3D image, and stereophotogrammetry [25].

**Figure 9:**
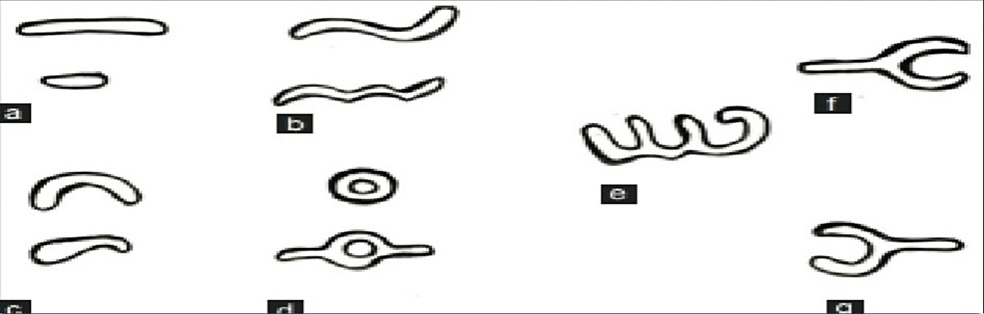
Diagram illustrating palatal rugae patterns: (a) linear, (b) sinusoidal, (c) curvilinear, (d) circular, (e) nonspecific, (f) diverging unification, and (g) converging unification

**Figure 10:**
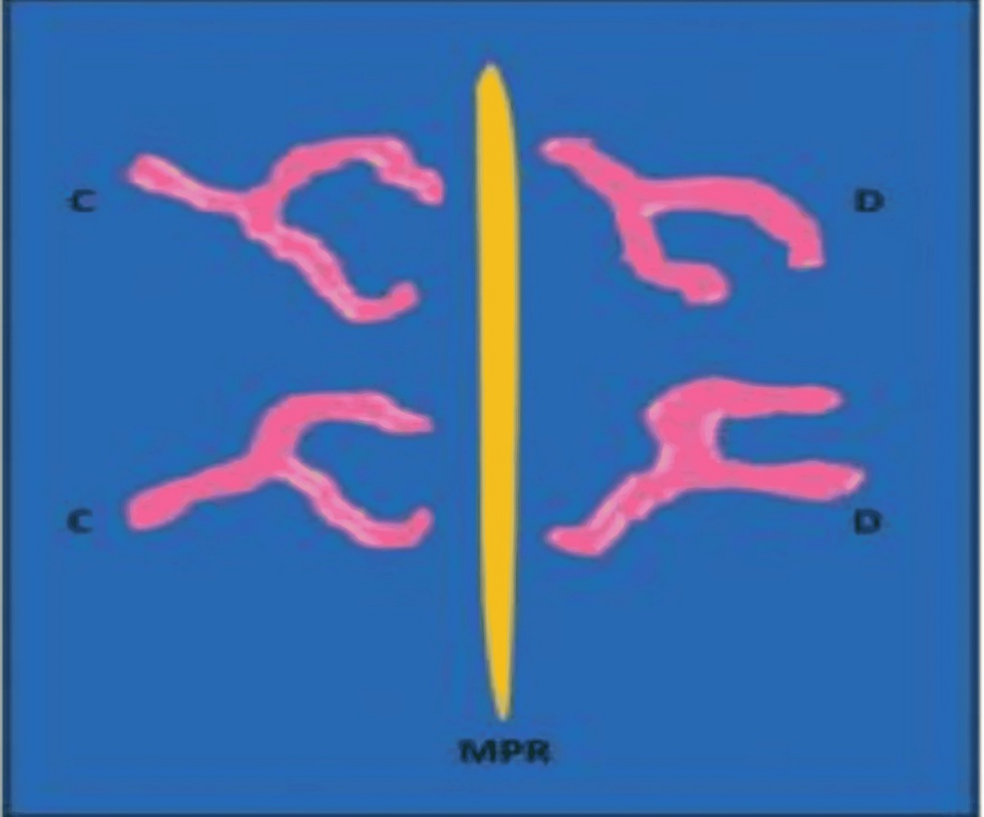
Palatal rugae unification patterns, including divergence and convergence

Rugae patterns were categorized according to their distribution along the sides, unification pattern, shape, palate direction, and primary-ruga length (Figures 9-10).

The dimension of the rugae, from start to end, was assessed, and they were categorized according to their length as follows: primary (rugae with a linear dimension greater than five millimeters), secondary (rugae with a linear dimension between three to five millimeters), and fragmentary (rugae with a linear dimension between two to three millimeters. Rugae with a linear dimension of less than two millimeters were excluded. Rugae shapes were identified as follows: curved (rugae falling into this category exhibit a curvilinear shape, including those that bend slightly at the origin or termination), wavy (with a sinusoidal pattern is termed as wavy), straight (designated as straight following a linear course from origin to termination), branched (classified as branched originate near the mid-palatine raphe and either bifurcate or unify before lateral termination), and circular (forming an uninterrupted curved ring is categorized as circular).

Rugae patterns are classified as follows: convergent pattern (rugae originating separately but merging along their lateral path) and divergent pattern (two rugae with a common origin bifurcating laterally in their course direction). Orientation was assessed by measuring the angle between the origin and termination axis of each ruga and the perpendicular to the raphe of the mid-palatine. Rugae were classified as forwardly positioned (positive angles), backwardly (negative angles), and zero degrees (perpendicular to the raphe of the median palatine) [22].

DNA Identification

DNA, the genetic code for organisms, is extracted from teeth using polymerase chain reaction (PCR), providing valuable material for identification tests. Saliva, collected non-invasively, is a robust DNA resource. Teeth offer genomic DNA even after extended postmortem periods. PCR enables the amplification of degraded DNA in routine forensic investigations. DNA from teeth is compared with ante-mortem samples like hair or epithelial cells and can be matched with the DNA of parents or siblings for identification [26].

DNA profiling is crucial when traditional forensic methods are unavailable, especially in cases with severely mutilated or decomposed remains where soft tissue is inaccessible. Teeth, protected by bone and soft tissue, serve as viable alternative sources for DNA isolation, offering better preservation and accessibility. Developing cost-effective, reliable, and less variable DNA methods is essential. Currently, DNA analysis is a reliable and less controversial tool in legal proceedings. Teeth play a significant role as a DNA source in forensic or investigative genetics [27].

Dental calculus proves to be an excellent reservoir for DNA for several reasons. Warinner et al. have reported dental calculus to be the richest known source of DNA. Dental calculus deposits are abundant and frequently observed in the oral cavity. Calculus can function as a DNA reservoir when destructive analysis of teeth or bones is prohibited. In cases where skeletal/dental remains are either unavailable or poorly preserved, calculus can still serve as a DNA reservoir. Due to its dense mineralization, calculus preserves biomolecules and microdebris over extended periods [28].

Identifying Blood Groups

This is crucial in the forensic sciences. This study demonstrates that DNA retrieval from human teeth is feasible for forensic cases, using the mentioned base pairs as a standard for ABO genotyping from pulp, as confirmed in this research [29].

Bite Mark Analysis

Bite marks are documented using photos or impressions, capturing tooth irregularities on the bitten surface. Create an accurate impression with materials like vinyl, polysiloxane, or polyether, applying more pressure for detail. Use acrylic or plaster as a rigid support for the impression to record skin curvature. Prosthodontist-taken bite records serve as valuable ante-mortem records, aiding comparison with postmortem findings. Physical characteristics like tooth spacing, arch shape, wear patterns, missing teeth, distance from canine to canine tips, and number of teeth marks are considered when comparing bite mark wounds. Capturing bite marks is challenging due to rapid shape and clarity changes (10-20 min) on the skin of the victims in both living and deceased individuals, necessitating instant recording. Despite prompt photography, 3D bite marks on a 2D image may alter color and spatial relations. Incomplete bite marks lack conclusiveness, requiring at least four to five teeth for reliable analysis [30].

Bite marks, often linked to assaults, sex crimes, and child abuse, display distinct patterns and may be found at theft scenes. Analyzing bite marks poses challenges, including a brief recording window, incomplete bites, and reduced reliability with fewer teeth. Identification entails assessing injury type, incident/crime scene details, site, individual tooth characteristics, color, size, and shape using photographs (2D and 3D), impressions, and digital impressions. Forensic departments encounter challenges in analysis and comparison due to tissue flexibility, jaw movements, impression distortion, faulty photographs, and various combinations. There are seven types of bite marks, as outlined in reference: hemorrhage (a tiny bleeding spot), contusion (bruise), artifact (a bitten-off piece), abrasion (a non-damaging mark on the skin), avulsion (skin removal), incision (torn skin), and laceration (a near puncture of the skin) [31].

Marks of bites on human skin tissues often occur in violent incidents like child abuse and sex crimes. These marks can result from the attacker biting the victim or vice versa in cases of self-defense, with the bite victim potentially being the suspect. Male victims are typically bitten on the shoulders and arms, while females are commonly bitten on the legs, arms, and breasts. Every group of teeth has distinct biting surfaces based on their function. Essential information, including demographics, shape, location, color, size, swabs, and type of injury, should be gathered from the bite victim. When gathering evidence, obtaining proper consent, details of the intra- and extra-oral examination, impressions of the lower and upper arches, photographs, and a detailed history are crucial. The comparison of bite marks involves analyzing and measuring the size, shape, and position of individual teeth [32].

Cameroon has created a detailed classification system for bite marks, grouping them into the materials involved and agents (humans, mammals, reptiles, and animals). Forensic odontologists, during bite mark analysis, compare the characteristics of the suspected individual's dentition with the bite mark pattern. Bite marks are vital and individualistic parameters used by forensic experts in criminal case resolution [33].

Digitization in forensic odontology: The use of digitization in forensic odontology is revolutionizing traditional investigative methods. Digital forensics enhances reliability in image analysis, reduces errors, and safeguards against manipulation. Implementing effective and legal software practices is vital for improved forensic investigations. Advances like genotyping bacteria and salivary DNA recovery from bite marks are becoming essential in this field. Digital forensics is on track to become a key element in investigations, with decreasing technology costs facilitating widespread adoption across specialties [34].

Numerous methods exist for analyzing bite mark patterns, and five distinct techniques were employed to create overlays by outlining the anterior teeth (mandibular and maxillary) on an acetate sheet. These techniques include the hand tracing technique (tracing was performed directly from study casts, with the acetate sheet placed on the surface of the bite of the lower and upper anterior teeth) and the radiographic wax impression technique (amalgam powder that is mixed with surgical spirit was applied to tooth impressions, and a radiographic image was captured on dental X-ray film). The resulting bite marks were then traced on a transparent sheet. Wax impression technique (impressions were taken on modeling wax sheets, and the resulting imprints were traced onto an acetate sheet) and xeroradiographic technique (lower and upper study casts were photocopied on an A4 sheet of paper with incisal edges down). The photocopy image was then overlaid with an acetate sheet, and the incisal edges were traced. 2D Computer Layout: The casts for the study were scanned on a 2D scanner, and a color photograph was generated. The image was transferred into Adobe Photoshop and rotated to align with the computer's x-axis, and the biting edges were highlighted using semi-automatic thresholding with the magnetic lasso tool. After initial selections, the chosen edges were smoothed and marked for comparison [35].

Radiographs and Imaging Techniques

Radiographs are important for identifying human remains, especially with adequate ante-mortem records. Forensic experts (odontologists, anthropologists, and dentists) compare pre- and post-mortem records using various intraoral and extraoral radiographs (e.g., orthopantomograms, skull views, CT scans, and cone beam CT (CBCT) scans). This is crucial when the mouth can't be opened. A portable X-ray unit is used at crime scenes to avoid disturbing teeth during body movement. Advanced radiographic tools reveal unique dental and skeletal features, facilitating the straightforward identification of victims or subjects [36].

CBCT has demonstrated effectiveness in procedures for forensic craniofacial reconstruction, utilizing standard milestones for facial soft and hard tissues. This technology proves valuable across various forensic applications, including stature assessment, age estimation, race and sex determination, cheiloscopy, analysis of bite marks, and more within the forensic department [37].

Forensic Profiling Using Salivary Signature

Saliva has emerged as a crucial tool in forensic investigations, playing a key role in detecting various crimes such as sexual assaults, human and animal bite marks, poisoning, hormone identification, and substance abuse. The potential of a salivary signature in forensics can be realized with improvements in the techniques for collecting and storing saliva. Additionally, it is essential to establish oral microbiome and salivary biomarker databanks specific to different populations and locations. This initiative would enable comparative analyses for both geological and personal identification purposes [38]. Saliva, easily obtained through noninvasive methods, comprises secreted products from plasma and salivary acini. Various identification tools are employed for screening, presumptive analysis, and confirmation of salivary constituents, proving valuable in both ante-mortem and postmortem assessments. This emerging research field requires further exploration to maximize its potential as a supplementary and confirmatory source for personnel profiling and identification [39].

Pediatric Forensic Dentistry

Pediatric dentistry, a specialized field, focuses on treating dental diseases in children. In forensics, it aids in identifying individuals through the visual, clinical, and radiographic interpretation of dental factors like the type and materials of restorations, any appliances, oral pathologies, syndromes, teeth condition, fractures, eruption and shedding sequences, and more. It also plays a crucial role in age estimation studies and recognizing instances of child abuse [40].

Age Estimation

Age estimation is crucial in forensic odontology for victim identification. Parameters like the estimation of dental age and measurements of anthropometric and skeletal maturation play a role. Among these, teeth are the most reliable for age estimation. Clinical, radiological, and histological techniques are used with dental landmarks at three intervals: (i) prenatal, neonatal, and early postnatal; (ii) children and adolescents; and (iii) adults. Clinical assessment evaluates tooth presence, visual changes, and periodontal status. Radiographic methods, both practical and nondestructive, aid in age determination for both living and deceased individuals by assessing crown and root formation stages. Histological approaches, determining enamel and dentin thickness, and analyzing dentin deposition and incremental lines contribute to age prediction. Pathologic age considers tissue alterations due to conditions, diseases, and processes, examining factors like root transparency and tooth structure wear. Physiologic age relates to growth and development changes. Investigators primarily focus on chronologic age- the time from birth to death. Forensic dentists use estimates of pathologic and physiologic age to assess the most likely chronologic age at the time of death [41].

Harvey identifies several factors for dental age estimation, including tooth germ appearance, early mineralization traces, unerupted tooth completion, enamel formation rate, and the presence of the neonatal line. Additional factors cover clinical eruption, erupted tooth root completion, deciduous tooth resorption, formation of secondary dentin, crown attrition, cementum formation, transparency of root dentin, resorption of the root surface, gingival recession, tooth discoloration, disease impact, alteration in chemical composition, sex influence, and malnutrition on tooth eruption [42].

For individuals in the stages of childhood, adolescence, and young adulthood, determining age becomes crucial in addressing issues related to criminal responsibility, child labor regulations, adoption, illegal immigration, reaching the age of majority, and eligibility for marriage, especially in the absence of a birth certificate. Orthodontic records, comprising photographs, radiographs, chronological age, anatomical age, dental age, and dental casts, offer valuable information for identification purposes. These records can reveal significant morphological, therapeutic, and pathological dental markers [43].

Sex Estimation

Forensic odontology is crucial in identifying the sex of individuals, particularly in cases of severe mass disasters with unrecognizable bodies. Dentists collaborate with experts, analyzing teeth and skulls for sex determination. Distinctive features in teeth, such as root length, morphology, and size of crown vary between female and male sexes. Skull patterns also differ, aiding in sex identification. Advanced techniques like PCR amplification enhance precise sex determination in remains [44]. Sex identification involves assessing the size, shape, and form of teeth. Frush and Fisher's, dentogenic concept highlights masculine and feminine characteristics that influence tooth shape in males and females [9]. Forensic scientists often employ odontometrics for sex determination. Additionally, specific bone features, such as the acute gonial angle of the mandible in females, help differentiate between males and females [45].

Methods for Sex Determination

The visual or clinical method: for tooth size, studies reveal notable differences in crown dimensions between male and female permanent and deciduous teeth. Mandibular canines exhibit the greatest dimensional disparity, with males having larger teeth. Quick and easy identification of sex can be achieved using permanent maxillary central incisors and mandibular mesiodistal dimensions. For root length and crown diameter, utilizing optical scanning and radiogrammetric measurements on mandibular permanent teeth. For canine dimorphism, Anderson and Thompson's study demonstrates greater mandibular canine width and inter-canine distance in males, enabling a 74% correct classification of sex. For dental index, Aitchison's "incisor index" (Ii) considers tooth proportions, especially the maxillary lateral incisor and central incisor. Higher Ii values in males support the notion that the lateral incisor is notably smaller than the central incisor in females. For odontometric differences, Işcan and Kedici highlight challenges in accurately diagnosing sex due to overlap in male and female tooth dimensions. Success is improved when considering all the available teeth. For tooth morphology, the distal accessory ridge, a nonmetric feature on the canine, is the most sexually dimorphic crown trait. Males exhibit significantly higher frequencies and more pronounced expression than females [46-47].

Microscopic Methods

Sex determination using Barr bodies: Analyzing X and Y chromosomes in non-dividing cells is a method for sex determination that is observable in various tissues. The X chromatin, known as the Barr body, is located near the nuclear membrane in females. Barr bodies and F bodies Y chromosomes, discovered by Barr et al. in 1950, can be preserved in dehydrated pulp tissues for up to one year. Pulp tissues retain sex diagnostic characteristics even when heated to 100°C for one hour [48].

Advanced Methods for Sex Determination

PCR, Sivagami et al. achieved 100% success in determining sex by preparing DNA from teeth through ultrasonication and subsequent PCR amplification. Enamel protein analysis and sex determination can be achieved by analyzing the amelogenin (AMEL) gene, which encodes for female amelogenin on the X chromosome and male amelogenin on the Y chromosome. Females have two identical AMEL genes, while males have two different AMEL genes. This method is effective for sex determination, even with very small DNA samples [49].

Recommendation

The creation of dedicated forensic odontology departments within dental institutes and the incorporation of forensic odontology courses as distinct programs sanctioned by the relevant dental council are suggested. These courses could be structured similarly to other dental specialties, allowing practitioners to specialize in forensic dentistry. Alternatively, postgraduate diploma courses, certificate courses, or short-term programs in forensic dentistry could be initiated. It is imperative for the dental education system to align training in forensic odontology with the evolving demands of the dental profession. This can be achieved by establishing specialized departments for forensic odontology within dental institutes.

## Conclusions

Forensic odontology stands as a promising and evolving field within dentistry, offering significant opportunities for further development. Forensic odontologists play a crucial role in crime scene investigations, particularly in interpreting dental evidence. The success of forensic dentistry hinges on the meticulous maintenance of ante-mortem records by dental specialists and institutions. These records should encompass essential information such as the individual's name, age, sex, tooth count, dental restorations, dentures, and morphological variations in teeth and mucosa, supported by photographs and radiographs. This comprehensive ante-mortem record becomes instrumental in the identification of deceased individuals and criminals when compared with postmortem records prepared during investigations, especially in cases of homicide and mass disasters. Given the remarkable resistance of teeth to destruction and decomposition, dental identification techniques, such as denture labeling, cheiloscopy, radiographs, bite mark analysis, rugoscopy, salivary signature, digitization in forensic odontology, age estimation, sex determination, dental DNA identification, implant analysis, and individual characteristics, can be applied effectively to both living and deceased persons, even in challenging conditions.
